# Supplementary figures and images for: Conditional ablation of heparan sulfate expression in stromal fibroblasts promotes tumor growth in vivo
Source: PLoS One. 2023 Feb 21;18(2):e0281820. doi: 10.1371/journal.pone.0281820 (PMC9942975; doi:10.1371/journal.pone.0281820)

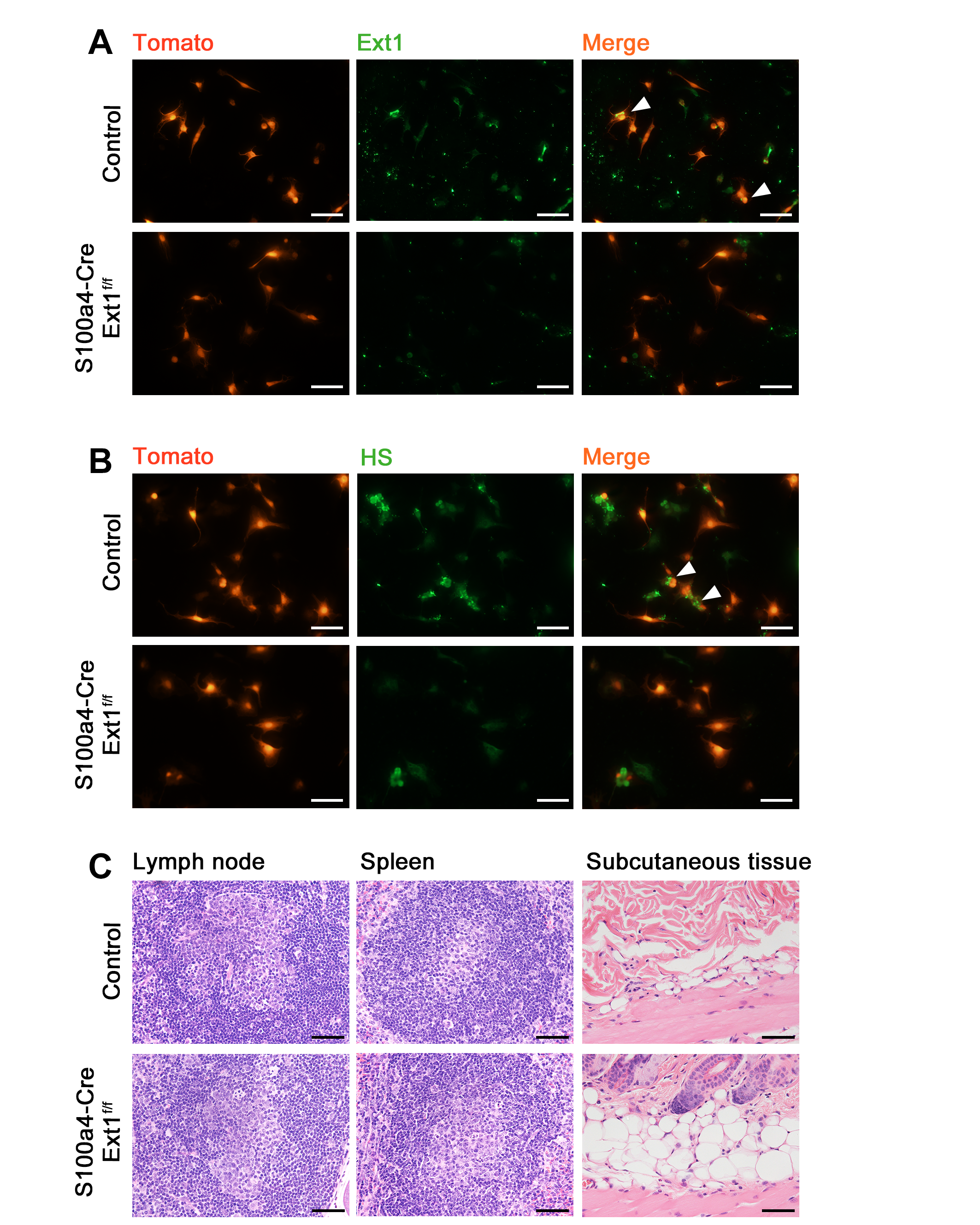

Supplement: S1 Fig — (A). Immunostaining for Ext1 in fibroblasts isolated from S100a4-Cre; Ext1f/f; Lsl-tdTomato and control (S100a4-Cre;Lsl-tdTomato) mice. Ext1 expression was reduced in fibroblasts of S100a4-Cre; Ext1f/f mice. White arrowheads indicate Ext1-positive fibroblasts. Scale bar = 50 μm. (B) Immunostaining for heparan sulfate (HS) in fibroblasts isolated from S100a4-Cre; Ext1f/f, Lsl-tdTomato, and control (S100a4-Cre;Lsl-tdTomato) mice. In control mice, the fibroblasts showed characteristic dot-like positive staining. White arrowheads indicate HS-positive fibroblasts. Scale bar = 50 μm. (C) Representative H&E staining of lymph node, spleen, and subcutaneous tissue of the skin in S100a4-Cre; Ext1f/f and control mice. Scale bar = 50 μm. (TIF) [file pone.0281820.s001.tif]

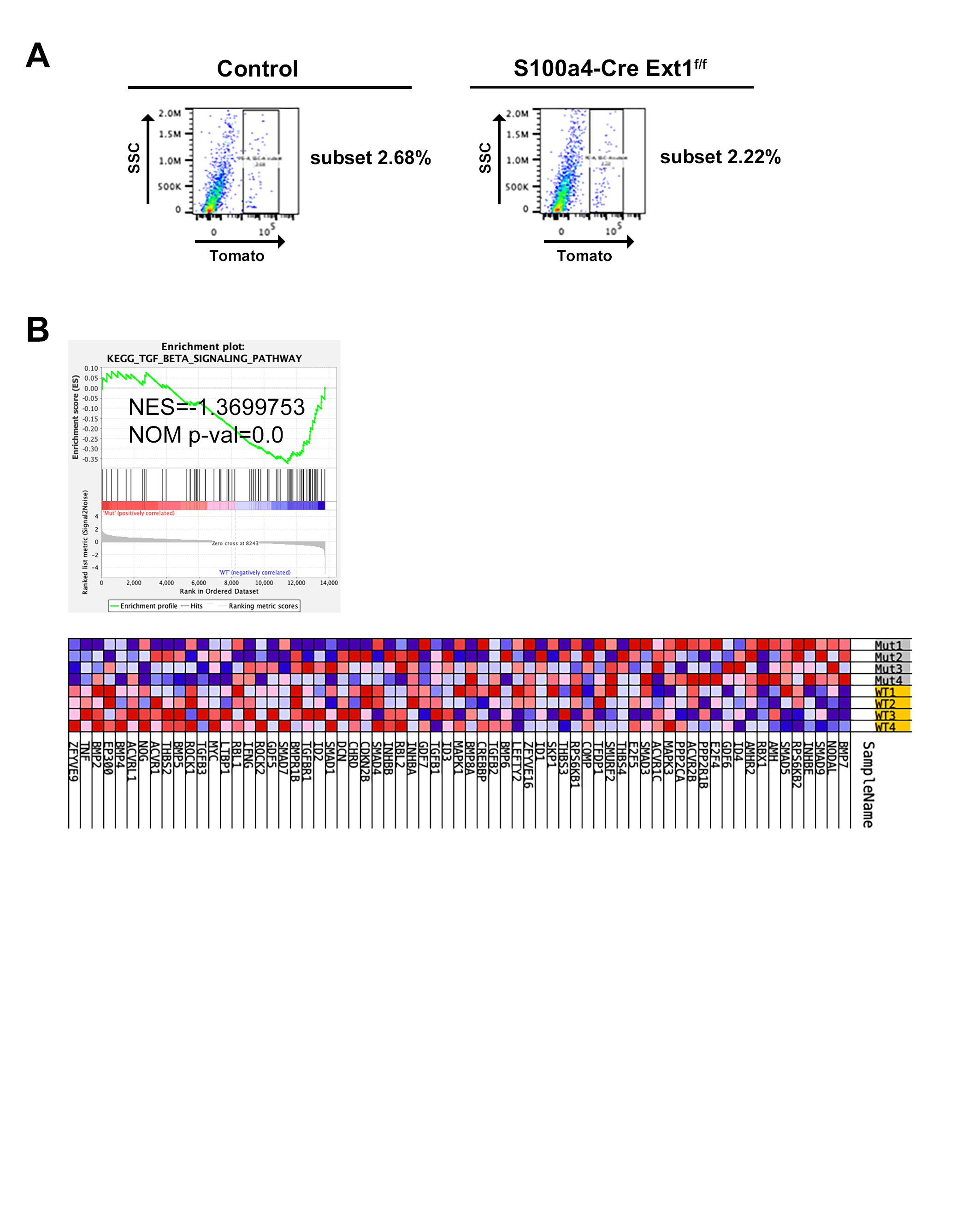

Supplement: S2 Fig — (A) Flow cytometric properties of the peri-tumor region of MC38 S.C. tumor in S100a4-Cre; Ext1f/f; Lsl-tdTomato, and control (S100a4-Cre; Lsl-tdTomato) mice (representative data). The subset represents Tomato-positive fibroblast cells. SSC, side scatter. (N = 3 for each cohort) (B) Gene set enrichment analysis (GSEA) obtained from microarray analysis of MC38 S.C. tumor of S100a4-Cre; Ext1f/f and control mice. MC38 S.C. tumors in S100a4-Cre; Ext1f/f mice showed decreased expression of a set of genes related to TGF-β signaling compared to control mice. WT means control mice, and Mut means S100a4-Cre; Ext1f/f mice (N = 4 for each cohort). (TIF) [file pone.0281820.s002.tif]

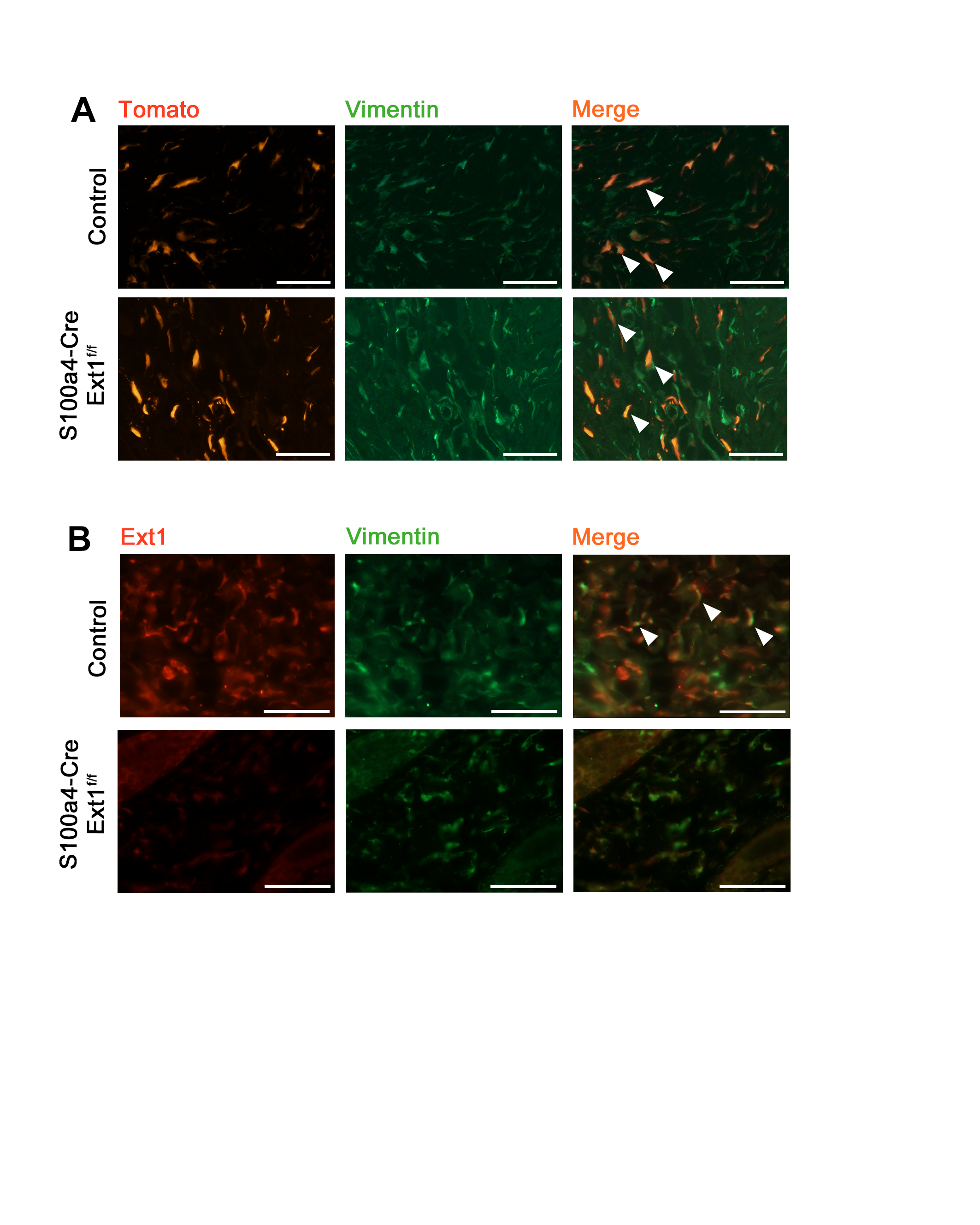

Supplement: S3 Fig — (A) Immunostaining of vimentin in subcutaneous tissues of the skin of S100a4-Cre; Ext1f/f, Lsl-tdTomato, and control (S100a4-Cre; Lsl-tdTomato) mice. White arrowheads indicate vimentin-positive fibroblasts. Scale bar = 50 μm. (B) Double immunofluorescence of Ext1 and vimentin in subcutaneous tissues of the skin of S100a4-Cre; Ext1f/f and control (Ext1f/f) mice. White arrowheads indicate Ext1 and vimentin double positive fibroblasts. Scale bar = 50 μm. (TIF) [file pone.0281820.s003.tif]

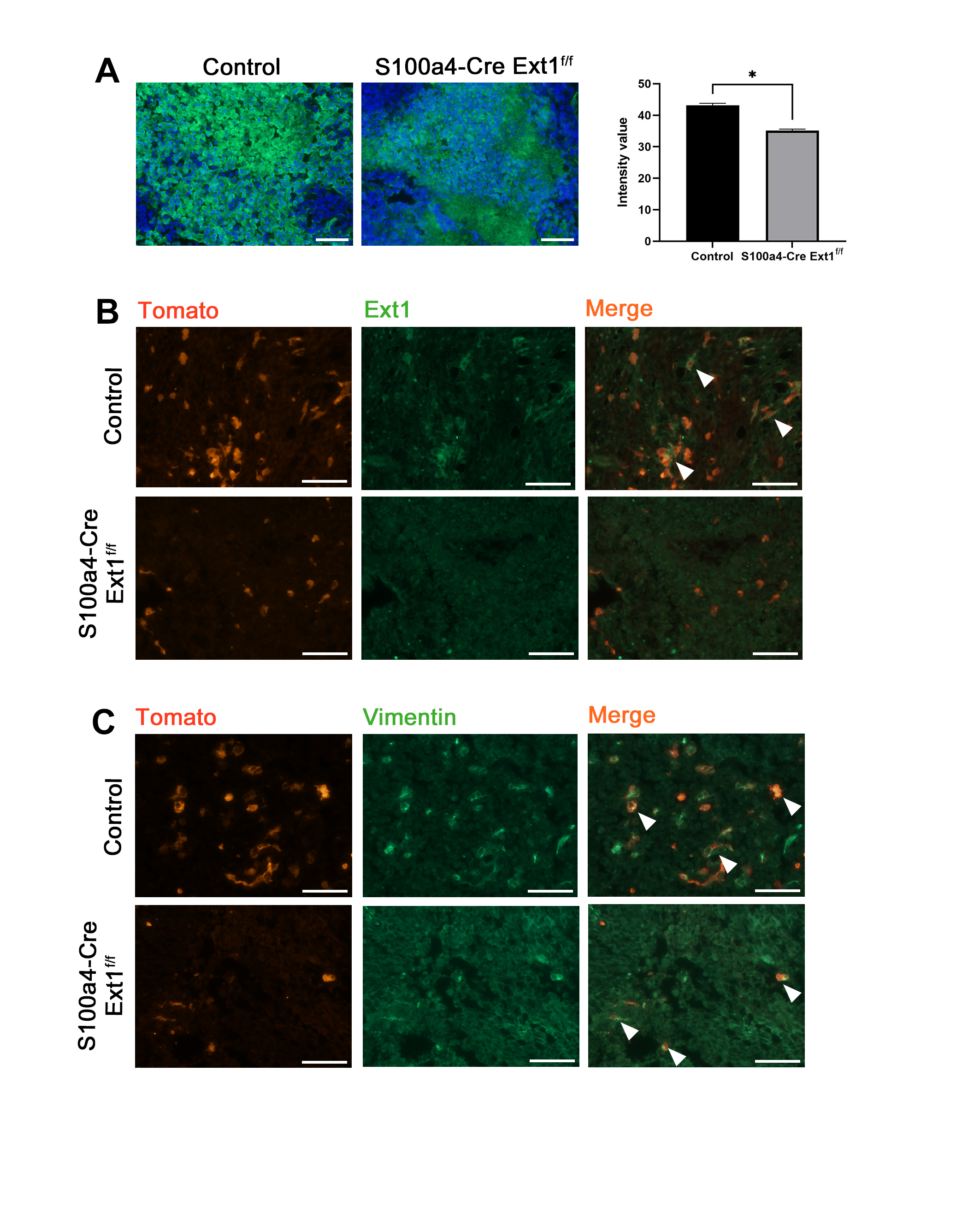

Supplement: S4 Fig — (A) Immunostaining of HS in MC38 S.C. tumor of S100a4-Cre; Ext1f/f; Lsl-tdTomato, and control (S100a4-Cre; Lsl-tdTomato) mice (left). The intensity value of HS stain in tumors of S100a4-Cre; Ext1f/f; Lsl-tdTomato, and control (S100a4-Cre; Lsl-tdTomato) mice (right). Data represent mean ± SEM. (N = 4 for each cohort, Mann–Whitney test, *P < 0.05). Scale bar = 50 μm. (B) Immunostaining of Ext1 in MC38 S.C. tumor of S100a4-Cre; Ext1f/f; Lsl-tdTomato, and control (S100a4-Cre; Lsl-tdTomato) mice. White arrowheads indicate Ext1-positive fibroblasts. Scale bar = 50 μm. (C) Immunostaining of vimentin in MC38 S.C. tumor of S100a4-Cre; Ext1f/f; Lsl-tdTomato, and control (S100a4-Cre; Lsl-tdTomato) mice. White arrowheads indicate vimentin-positive fibroblasts. Scale bar = 50 μm. (TIF) [file pone.0281820.s004.tif]

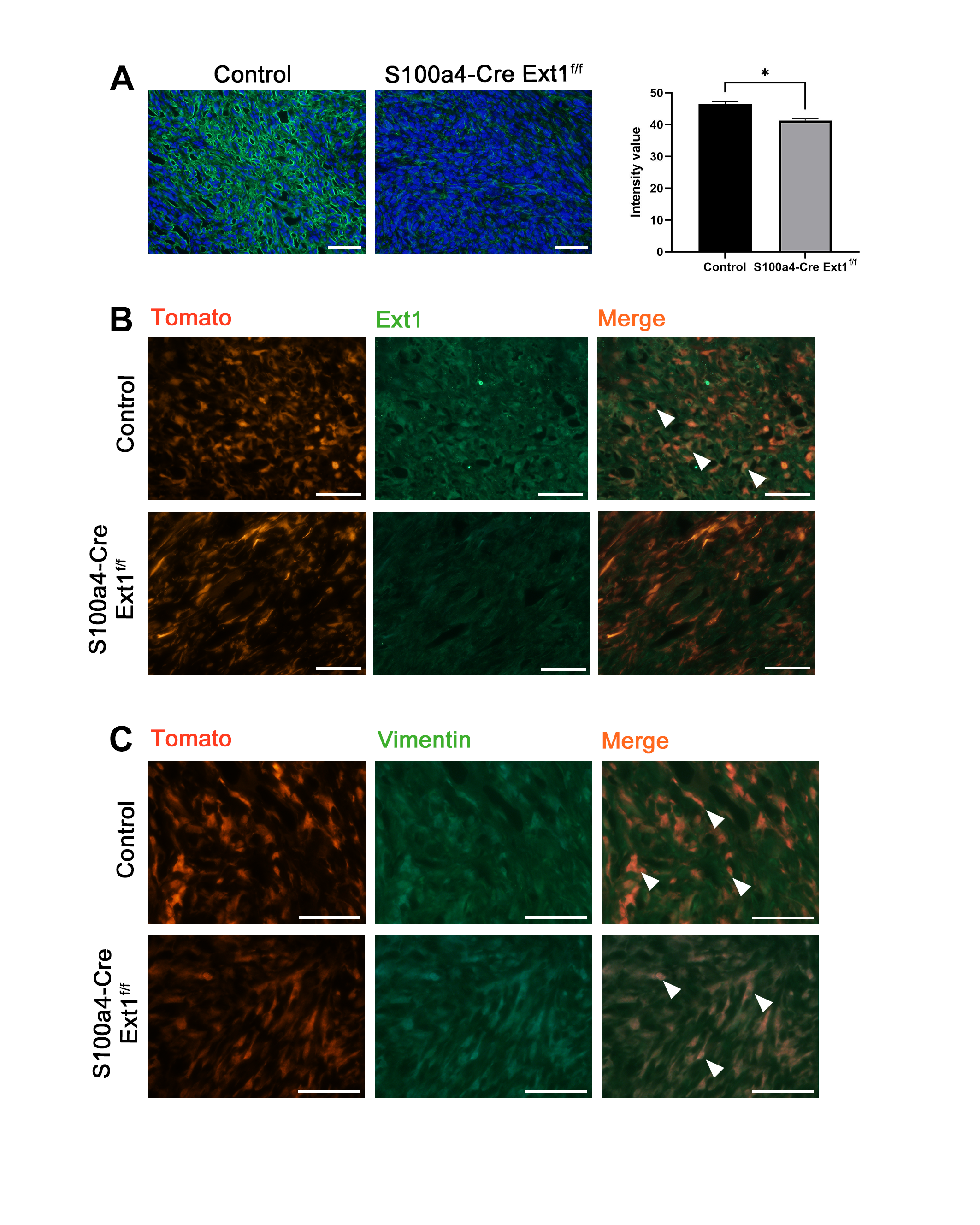

Supplement: S5 Fig — (A) Immunostaining of HS in Pan02 S.C. tumor of S100a4-Cre; Ext1f/f; Lsl-tdTomato, and control (S100a4-Cre; Lsl-tdTomato) mice (left). The intensity value of HS stain in tumors of S100a4-Cre; Ext1f/f; Lsl-tdTomato, and control (S100a4-Cre; Lsl-tdTomato) mice (right). Data represent mean ± SEM. (N = 4 for each cohort, Mann–Whitney test, *P < 0.05). Scale bar = 50 μm. (B) Immunostaining of Ext1 in Pan02 S.C. tumor of S100a4-Cre; Ext1f/f; Lsl-tdTomato, and control (S100a4-Cre; Lsl-tdTomato) mice. White arrowheads indicate Ext1-positive fibroblasts. Scale bar = 50 μm. (C) Immunostaining of vimentin in Pan02 S.C. tumor of S100a4-Cre; Ext1f/f; Lsl-tdTomato, and control (S100a4-Cre; Lsl-tdTomato) mice. White arrowheads indicate vimentin-positive fibroblasts. Scale bar = 50 μm. (TIF) [file pone.0281820.s005.tif]

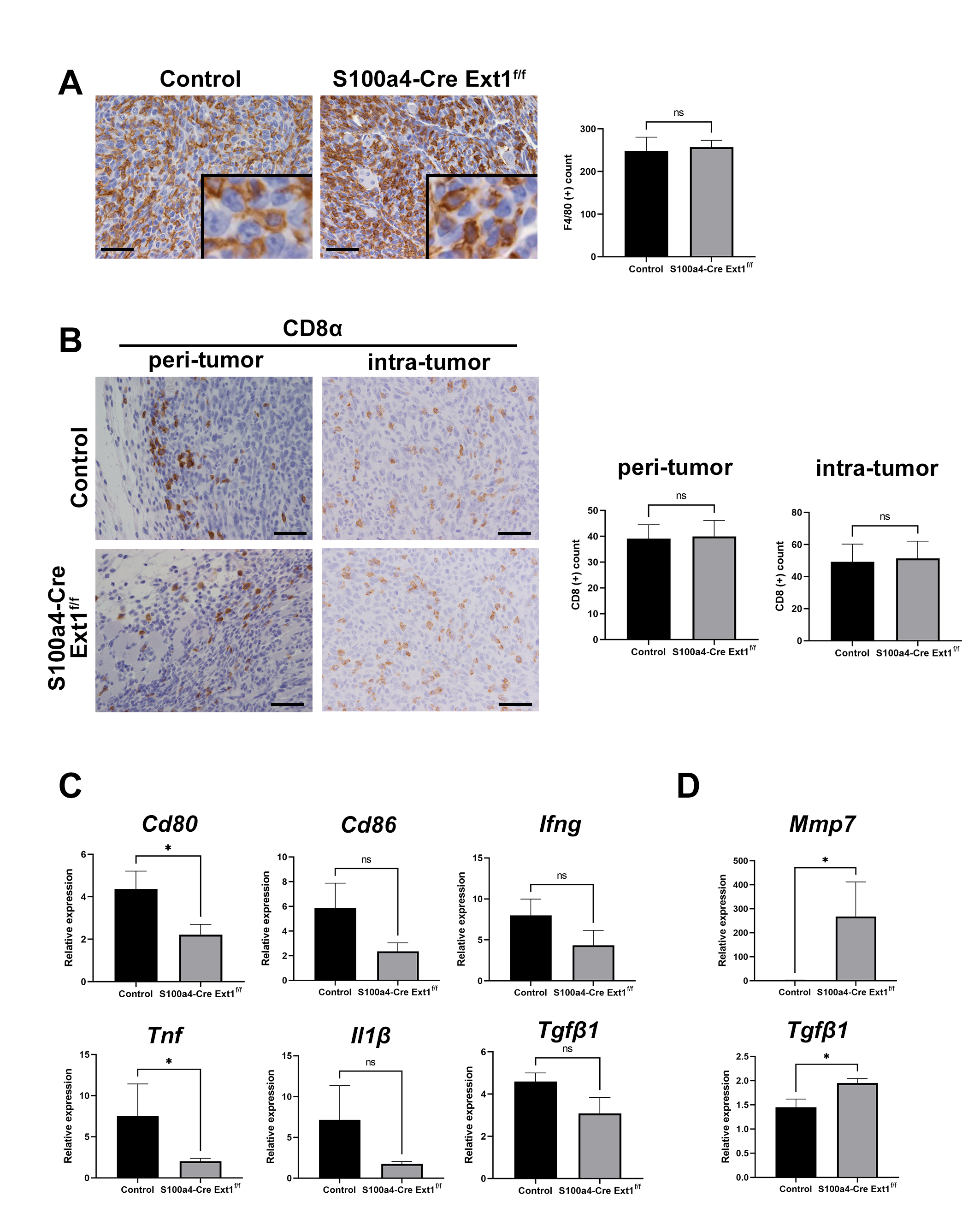

Supplement: S6 Fig — (A) Immunohistochemistry for F4/80 of Pan02 S.C. tumor of S100a4-Cre; Ext1f/f and control mice (left). The number of F4/80-positive cells in tumors of S100a4-Cre; Ext1f/f and control mice (right). Data represent mean ± SEM (N = 6 for each cohort, unpaired t-test). Scale bar = 50 μm. (B) Immunohistochemistry for CD8α in peri- and intra-tumor regions of Pan02 S.C. tumors of S100a4-Cre; Ext1f/f and control mice (left). The number of CD8α-positive cells in peri- and intra-tumor regions of tumors of S100a4-Cre; Ext1f/f and control mice (right). Data represent mean ± SEM (N = 6 for each cohort, Mann–Whitney test). Scale bar = 50 μm. (C) Quantitative real-time RT-PCR analysis of genes related to the immune microenvironment in MC38 S.C. tumors of S100a4-Cre; Ext1f/f and control mice. Data represent mean ± SEM (N = 4 for each cohort, Mann–Whitney test, *P < 0.05). (D) Quantitative real-time RT-PCR analysis of Mmp7 and Tgfβ1 in Pan02 S.C. tumors of S100a4-Cre; Ext1f/f and control mice. Data represent mean ± SEM (N = 4 for each cohort, Mann–Whitney test [Mmp7] and unpaired t-test [Tgfβ1], *P < 0.05). (TIF) [file pone.0281820.s006.tif]

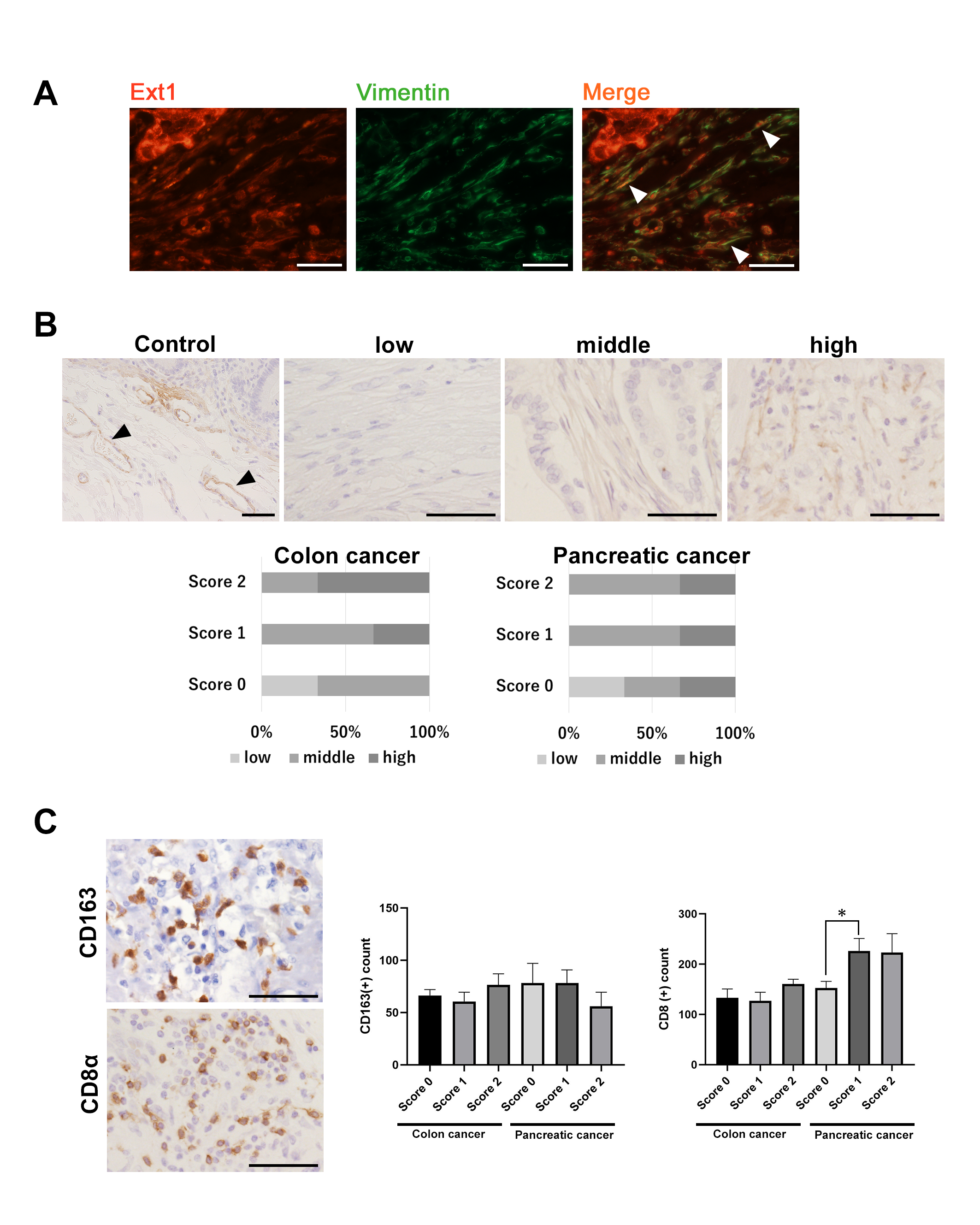

Supplement: S7 Fig — (A) Double immunofluorescence of Ext1 and vimentin in human cancer stroma. White arrowheads indicate Ext1 and vimentin double-positive fibroblasts. Scale bar = 50 μm. (B) Immunostaining of αSMA in human cancer stroma (top). Arrowheads indicate that the vascular smooth muscle showed αSMA-positive as a positive control. Scale bar = 50 μm. Expression level of αSMA in human colon and pancreatic cancers of each Ext1 score (N = 3 each score) (bottom). (C) Immunostaining of CD163 and CD8α in human cancer stroma (left). Scale bar = 50 μm. The number of CD163- and CD8α-positive cells in human colon and pancreatic cancers of each Ext1 score (N = 3 each score) (right). Data represent mean ± SEM (N = 3 for each cohort, Mann–Whitney test, *P < 0.05). (TIF) [file pone.0281820.s007.tif]
