# Supplementary material for: Conditional ablation of heparan sulfate expression in stromal fibroblasts promotes tumor growth in vivo
Source: PLoS One. 2023 Feb 21;18(2):e0281820. doi: 10.1371/journal.pone.0281820 (PMC9942975; doi:10.1371/journal.pone.0281820)
Supplement: S1 Table — (DOCX) [file pone.0281820.s008.docx]

**Supplementary Table.1** Probe name of microarray data.

| *Gene Symbol* | *Probe name* |
| --- | --- |
| Cd80 | A_51_P418295 |
| Cd80 | A_52_P244005 |
| Cd80 | A_66_P130432 |
| Cd86 | A_51_P174723 |
| Cd86 | A_52_P145861 |
| Cd86 | A_55_P2715366 |
| Cd86 | A_55_P1971951 |
| Ifng | A_52_P68893 |
| Tnf | A_51_P385099 |
| Il1β | A_51_P212782 |
| Il1β | A_66_P114500 |
| Il1β | A_55_P2720183 |
| Mmp7 | A_51_P426096 |
| Mmp7 | A_66_P118835 |
