# Supplementary material for: Conditional ablation of heparan sulfate expression in stromal fibroblasts promotes tumor growth in vivo
Source: PLoS One. 2023 Feb 21;18(2):e0281820. doi: 10.1371/journal.pone.0281820 (PMC9942975; doi:10.1371/journal.pone.0281820)
Supplement: S2 Table — (DOCX) [file pone.0281820.s009.DOCX]

**Supplementary Table.2** Primer list of real-time RT-PCR data.

| *Mouse gene* | *Forward primer* | *Reverse primer* |
| --- | --- | --- |
| *Cd80* | 5’-TCAGTTGATGCAGGATACACCA-3’ | 5’-AAAGACGAATCAGCAGCACAA-3’ |
| *Cd86* | 5’-TCAATGGGACTGCATATCTGCC-3’ | 5’-GCCAAAATACTACCAGCTCACT-3’ |
| *Ifng* | 5’-GCCACGGCACAGTCATTGA-3’ | 5’-TGCTGATGGCCTGATTGTCTT-3’ |
| *Tnf* | 5’-CAGGCGGTGCCTATGTCTC-3’ | 5’-CGATCACCCCGAAGTTCAGTAG-3’ |
| *Il1β* | 5’-GAAATGCCACCTTTTGACAGTG-3’ | 5’-TGGATGCTCTCATCAGGACAG-3’ |
| *Tgfβ1* | 5’-CCACCTGCAAGACCATCGAC-3’ | 5’-CTGGCGAGCCTTAGTTTGGAC-3’ |
| *Mmp7* | 5’-CTTACCTCGGATCGTAGTGGA-3’ | 5’-CCCCAACTAACCCTCTTGAAGT-3’ |
