# Supplementary material for: Conditional ablation of heparan sulfate expression in stromal fibroblasts promotes tumor growth in vivo
Source: PLoS One. 2023 Feb 21;18(2):e0281820. doi: 10.1371/journal.pone.0281820 (PMC9942975; doi:10.1371/journal.pone.0281820)
Supplement: Attachment — Submitted filename: niwa_R2_response_letter.docx [file pone.0281820.s011.docx]

**Response letter**

**Author responses to reviewers**

**Reviewer #1:**

***The authors have addressed all of the three comments previously raised by this reviewer, which seemed to be all satisfactory. They truthfully showed that the dependence of tumor growth on HS might be different between tumor types, and this could be a useful information for us to consider inter-tumoral heterogeneity that is also relevant to human cases. I have no additional comments on the manuscript and recommend it for publication.***

**Response:** Thank you so much for your cooperation.

**Reviewer #2:**

***The revised manuscript has addressed many of my concerns, however, there are still a few issues that need to be addressed before publication.

1. In Supplementary 4C, the images of tdTomato in the control and S100a4-Cre Ext1 f/f groups appear to be identical and should be replaced with more appropriate images.***

**Response:** Thank you for your indication. We have changed it with the appropriate image. Please see revised Supplementary Fig. 4C.

***2. In Supplementary 5C, tdTomato and vimentin signals in S100a4-Cre Ext1 f/f are too perfectly matched, which is strange given that vimentin-negative cells also express S100a4, as shown in Figure 3A. More suitable images should be used.***

**Response:** Yes, we agree. We have changed and enlarged the figures to show vimentin-negative cells clearly. Please see revised Supplementary Fig. 5C.

***3. In Supplementary 6C and D, it is unclear how the relative expression values were calculated and relative to what value. Could the authors provide more information on this? Also, the data should be separated by each gene, not combined in one figure.***

**Response:** Thank you for your suggestion. To analyze relative gene expression, the comparative Ct method was used and relative values of gene expression levels were calculated by setting the expression level of the sample with the lowest expression level as 1. We have revised the Materials and methods in the manuscript (page 13, lines 15-18). We also changed the data for each gene separately. Please see revised Supplementary Figure 6C and D.
